# Healthcare resource utilization and costs associated with generalized myasthenia gravis: a retrospective matched cohort study using the National Health Insurance Research Database in Taiwan

**DOI:** 10.3389/fneur.2023.1216595

**Published:** 2023-07-26

**Authors:** Shih-Pei Shen, Keira Joann Herr, Yanfang Liu, Chih-Chao Yang, Chao-Hsiun Tang

**Affiliations:** ^1^School of Health Care Administration, College of Management, Taipei Medical University, Taipei, Taiwan; ^2^Janssen Medical Affairs Asia Pacific, Singapore, Singapore; ^3^Global Real-World Evidence, GCDS, GCSO, Janssen Research & Development LLC, Raritan, NJ, United States; ^4^Department of Neurology, National Taiwan University Hospital, Taipei, Taiwan

**Keywords:** myasthenia gravis, cost, healthcare resource utilization, Taiwan, employment

## Abstract

**Background:**

We estimated healthcare resource utilization (HRU) and costs in patients with generalized myasthenia gravis (gMG) in Taiwan.

**Methods:**

This retrospective population-based, matched cohort study used the National Health Insurance Research Database to identify prevalent patients with gMG (cases) in 2019. In total, 2537 cases were matched (1:4) by age, sex, and urbanization level to 10148 randomly selected patients without gMG (comparators). A generalized linear regression model predicted the frequency of HRU and costs among service users. Costs attributable to gMG were obtained by subtracting all-cause HRU costs incurred by comparators from cases.

**Results:**

The mean age of all patients was 54.99 years and 55.97% were female. Compared with comparators, cases had significantly higher rates of hypertension (33.03%/24.26%), diabetes mellitus (18.92%/11.37%), malignancies (16.00%/4.08%), cardiovascular disease (11.35%/8.12%), thyroid-related conditions (5.99%/1.16%), respiratory illness/disorders (4.38%/1.22%), and neurotic disorders (4.65%/2.6%). Amongst users of healthcare resources, cases had a mean 10 additional outpatient visits, 0.62 inpatient stays, and 0.49 emergency room visits in 2019 compared with comparators (*p* < 0.0001 for all). The mean (standard deviation) difference in all-cause healthcare costs between cases and comparators was NT$ 94997 (76431) [US$ 3133 (2521)], and was significantly higher for all categories (outpatient, inpatient, emergency room, drugs; *p* < 0.0001 for all). Among employed persons, 13.18%/7.59% of cases/comparators changed employment status during the study (*p* < 0.0001).

**Conclusion:**

gMG presents a substantial burden on HRU and healthcare costs in Taiwan. A high attrition rate from full-time employment suggests additional societal costs. Improved treatments are needed to alleviate the burden of disease on individuals, healthcare systems, and economies.

## 1. Introduction

Myasthenia gravis (MG) is a rare, chronic autoimmune disease caused by antibodies directed at acetylcholine receptors, muscle-specific kinases, or lipoprotein-associated protein at the neuromuscular junction (1). Patients commonly present with ocular weakness, and approximately 50% of patients develop generalized symptoms in the first 2 years following diagnosis (2, 3). Generalized MG (gMG) is characterized by fluctuating muscle weakness and fatigue that variably affects the oropharyngeal muscles, limbs, neck, and diaphragm (4). Patients can experience difficulty in speaking, swallowing, and mobilizing. Poor posture secondary to muscle weakness can cause chronic pain, and sleep may be disturbed due to respiratory muscle weakness (4). The most serious complication of gMG is myasthenia crisis, where extreme respiratory and bulbar muscle weakness require temporary intubation and mechanical ventilation. Myasthenia crisis affects 15%–20% of patients during their lifetime (5).

gMG requires life-long treatment with individualized combinations of anti-cholinesterase inhibitors, thymectomy when indicated, immunosuppression using corticosteroids and steroid-sparing agents such as azathioprine, mycophenolate mofetil, cyclosporine, cyclophosphamide, or methotrexate (6). Patients with severe or refractory disease may respond to intravenous immunoglobulin (IVIg), plasma exchange, or treatment with monoclonal antibodies such as rituximab or eculizumab (7).

A systematic review of the literature published until 2020 found that information about the costs associated with the treatment of MG was sparse, and that health resource utilization (HRU) and associated costs varied markedly between and within countries (8). Key drivers of the direct medical cost of the illness included the use of IVIg and plasma exchange, treatment of myasthenic crises including mechanical ventilatory support, and hospitalizations (8).

We previously reported the epidemiology of gMG in Taiwan using the population-based Taiwan National Healthcare Insurance Research Database (NHIRD). Between 2009 until 2019, the prevalence of patients with gMG in Taiwan increased significantly, from 6.83 per 100000 population to 11.18 per 100000 population (submitted), with an increasing proportion of older adults with gMG. We observed that the epidemiology of gMG in Taiwan is evolving rapidly, pointing to a growing burden of disease with associated increases in HRU and healthcare costs. In parallel, new treatments for gMG are becoming available, and information about HRU and healthcare costs will be needed to inform health technology assessments. In this study, we estimated HRU and costs associated with gMG by conducting a population-based study using the NHIRD. The costs associated with gMG were assessed by comparing outcomes in patients with and without gMG.

## 2. Methods

### 2.1. Data source

Taiwan’s National Health Insurance (NHI) program provides compulsory health insurance to 99.9% of the Taiwanese population of approximately 24 million (9). All healthcare services including outpatient and inpatient services, dental care services, traditional medicine, prescription drugs, and laboratory and imaging examinations are available to members. The NHI captures claim-based information that is released to the NHIRD which holds information on all medical services provided by NHI-contracted hospitals, physicians, and pharmacies to residents throughout Taiwan. The database holds demographic information, the type and date of services rendered, and diagnoses coded using International Classification of Diseases, Tenth Revision (ICD-10) codes. All members have a unique ID that enables all healthcare episodes to be captured regardless of where they occurred, and which can be linked to the Death Registry using scrambled identification numbers.

The NHIRD is provided by the NHI Center. All analyses used de-identified, aggregated patient data. Administration is maintained by the Data Science Centre of the Ministry of Health and Welfare in Taiwan. This study was conducted according to all applicable guidelines and regulations set by the Health and Welfare Data Science Center and was granted an exemption from ethical review by Taipei Medical University-Joint Institutional Review Board.

### 2.2. Study design and population

This retrospective population-based, matched cohort study evaluated HRU and costs from the payer’s perspective. The study cohort included prevalent patients with a diagnosis of MG (ICD-10 G70.00, G70.01) in the NHIRD in 2019. Patients with gMG were identified as having an inpatient claim with MG as the main or sub-diagnosis under any discipline (patients with an ophthalmologic diagnosis alone as the main or sub-diagnosis were excluded); and a prescription for azathioprine or at least one treatment period of steroids at a minimum dose of 20 mg prednisolone (or equivalent) daily for 28 days. Patients with gMG who died during 2019 were excluded from the analysis.

A comparator cohort of patients without gMG was identified by randomly selecting individuals with no claim for a diagnosis of MG during 2019 in the database. Four comparators were matched to each case by age, sex, and residential urbanization level (10). Costs were assessed from 01 January until 31 December 2019. Comparators who died during 2019 were replaced with another control.

### 2.3. Outcomes

The Charlson comorbidity index (CCI) was used for evaluating comorbidities using ICD-10 codes.

All-cause HRU and costs were captured for cases and comparators during 2019. Costs associated with outpatient, inpatient, and emergency department (ED) services were evaluated. Costs were further broken down by medication and non-medication costs. Non-medication costs included all fees for physician consultations, inpatient episodes, diagnostic and laboratory tests, procedures, and surgery. An estimate of the HRU and costs attributable to gMG was obtained by subtracting the all-cause HRU and costs incurred by patients with gMG from all-cause HRU and costs incurred by their matched comparators.

Costs are presented in New Taiwan dollars (NT$). In early 2023, 100 NT$ converts to around 3.0 Euros and 3.3 USD.

### 2.4. Statistical analysis

Continuous variables were described using means, standard deviations (SD), medians, and interquartile ranges (IQR). Categorical variables were described using frequencies and percentages. chi-square and *t*-tests were applied to assess potential differences between cases and comparators for categorical and continuous variables, respectively.

Raw mean all-cause HRU and costs in 2019 were reported for patients with gMG and comparators. To account for the potential over-representation of zeros and right-skewed distribution of HRU and cost data, a two-part model was performed: a logistic regression to predict the probability of service use and a generalized linear model to predict the frequency of HRU and costs among those with positive frequency of service. Covariates entered into the models to predict HRU and costs included age, sex, urbanization index, and comorbidities.

Cases/comparators who had a change in employment status during 2019, either becoming unemployed and dependent on their children/relatives, or who moved into a lower income employment insurance category, were identified. Cases/comparators who turned 65 during 2019 (the age of retirement from the NHI) were excluded from this analysis.

All statistical analyses were performed using SAS Version 9.4 (SAS Institute, Cary, NC, United States).

## 3. Results

**Figure 1:**
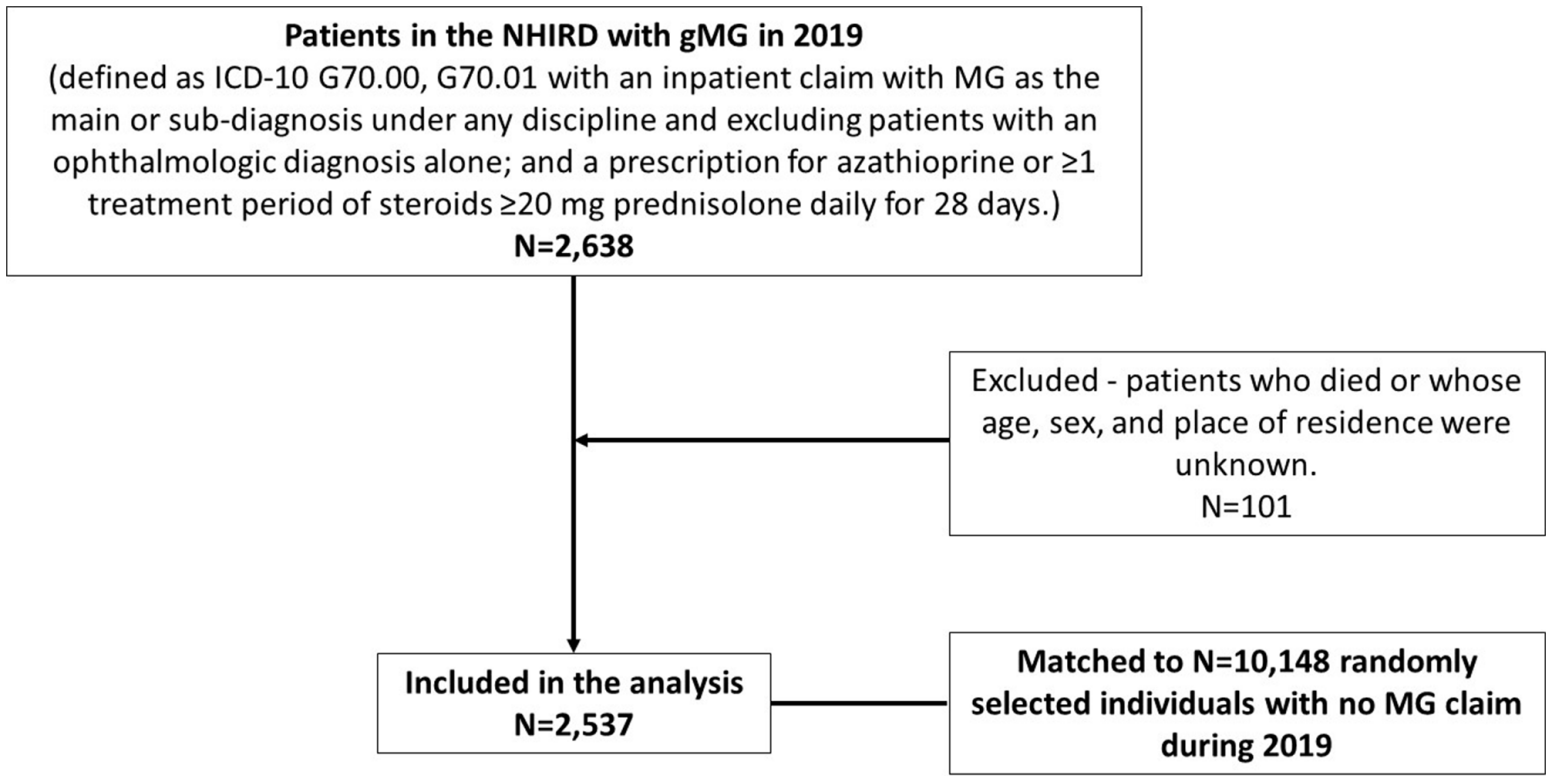
Patient flow.

There were 2537 prevalent patients (cases) with gMG in the NHIRD in 2019 matched to 10148 comparators (Figure 1). The mean age of patients was 54.99 years (SD 16.08), 55.97% were female, and 46.2% were between 50 and 69 years of age (Table 1). Most cases and comparators (84.0%) resided in areas of high urbanization (levels 1–4). Cases had a higher CCI than comparators [mean 1.26 (SD 1.74) vs. 0.59 SD (1.2)], and 32.44% of cases had a CCI score ≥2 versus 14.77% of comparators. Compared with comparators, cases had significantly higher rates of hypertension (33.03% vs. 24.26%), diabetes mellitus (18.92% vs. 11.37%), malignancies (16.00% vs. 4.08%), cardiovascular disease (11.35% vs. 8.12%), thyroid related conditions (5.99% vs. 1.16%), respiratory illness/disorders (4.38% vs. 1.22%), and neurotic disorders (4.65% vs. 2.6%).

**Table 1:** Demographic characteristics of patients with generalized myasthenia gravis and comparators during 2019 in Taiwan.

Category	Case (*n* = 2537)		Comparators (*n* = 10148)		*p*
	*n*	%	*n*	%	
Gender					1.0000
Male	1117	44.03	4468	44.03	
Female	1420	55.97	5680	55.97	
Age (years)					1.0000
≤9	9	0.35	36	0.35	
10–19	42	1.66	168	1.66	
20–29	111	4.38	444	4.38	
30–39	278	10.96	1112	10.96	
40–49	454	17.90	1816	17.90	
50–59	573	22.59	2292	22.59	
60–69	598	23.57	2392	23.57	
70–79	321	12.65	1284	12.65	
≥ 80	151	5.95	604	5.95	
Mean (±SD)	54.99	(16.08)	54.99	(16.08)	
Median (range)	56 (2, 99)		56 (2, 99)		
Urbanisation level of residential area					1.0000
1 (most urbanized)	680	26.80	2720	26.80	
2	870	34.29	3480	34.29	
3	268	10.56	1072	10.56	
4	313	12.34	1252	12.34	
5	127	5.01	508	5.01	
6	115	4.53	460	4.53	
7 (least urbanized)	164	6.46	656	6.46	
*CCI*					<0.0001
Mean (±SD)	1.26	(1.74)	0.59	(1.2)	
0	1144	45.09	7057	69.54	
1	570	22.47	1592	15.69	
2	413	16.28	832	8.2	
≥3	410	16.16	667	6.57	
*Comorbidities*					
Hypertension	838	33.03	2462	24.26	<0.0001
Diabetes mellitus: type I or II	480	18.92	1154	11.37	<0.0001
Malignancies	406	16.00	414	4.08	<0.0001
Cardiovascular disease	288	11.35	824	8.12	<0.0001
Thyroid related conditions	152	5.99	118	1.16	<0.0001
Respiratory illness/disorder	111	4.38	124	1.22	<0.0001
Neurotic disorder	118	4.65	264	2.6	<0.0001
Renal conditions	97	3.82	363	3.58	0.5527
Obesity	6	0.24	17	0.17	0.4651
Liver abnormalities	4	0.16	15	0.15	0.9086

CCI, Charlson comorbidity index; *N*, number of subjects; SD, standard deviation.

### 3.1. Health resource utilization associated with gMG

The majority of cases and comparators (99.92% and 94.79%) used outpatient services in 2019. Inpatient and ED episodes were recorded for 44.42% and 37.33% of cases, respectively, and 10.68% and 17.75% of comparators (Table 2). Among users of these services, cases had a mean of 27.51 (SD 17.19) outpatient episodes, versus 17.68 (SD 15.50) in comparators. The mean number of inpatient episodes was 1.77 (SD 1.44) among cases and 1.57 (SD 1.45) in comparators, and the mean number of ED episodes was 2.09 (SD 2.55) and 1.63 (SD 1.77), respectively.

**Table 2:** Annual raw health resource utilization and costs among patients with gMG and comparators.

	Cases (*n* = 2537)				Comparators (*n* = 10148)			
	Proportion of usage		Number of events in 2019 among non-zero users		Proportion of usage		Number of events in 2019 among non-zero users	
	*n*	%	Mean	SD	*n*	%	Mean	SD
*Frequency of care*								
Outpatient	2535	99.92	27.51	17.19	9619	94.79	17.68	15.50
Inpatient	1127	44.42	1.77	1.44	1084	10.68	1.57	1.45
Emergency	947	37.33	2.09	2.55	1801	17.75	1.63	1.77
*Costs of care (NT$)*								
Outpatient	2535	99.92	40325.74	69886.97	9619	94.79	19753.27	54133.81
Inpatient	1127	44.42	142419.62	214202.74	1084	10.68	88578.73	133801.87
Emergency	947	37.33	10326.42	14661.81	1801	17.75	6273.67	9910.83
Drugs	2537	100	27804.26	65976.59	9637	94.96	10051.68	45042.05
Total	2537	100	135219.23	224174.16	9637	94.96	40904.12	106625.26

CCI, Charlson comorbidity index; *N*, number of subjects; NT$, New Taiwan dollars; SD, standard deviation.

In the regression analysis, the frequency of care amongst users of healthcare resources was significantly higher in cases than comparators. Cases had a mean 10 additional outpatient visits, 0.62 inpatients stays, and 0.49 ED visits compared with comparators in 2019 (*p* < 0.0001 for all comparisons) (Table 3).

**Table 3:** Annual regression-adjusted heath resource utilization and associated costs among patients with gMG and comparators.

	Cases (*n* = 2537)		Comparators (*n* = 10148)		Difference		*p*-value
	Mean	SD	Mean	SD	Mean	SD	
*Frequency of care*							
Outpatient	27.51	7.22	16.93	9.05	10.57	8.71	<0.0001
Inpatient	0.79	0.52	0.17	0.28	0.62	0.34	<0.0001
Emergency	0.78	0.47	0.29	0.27	0.49	0.32	<0.0001
*Costs of care (NT$)*							
Outpatient	41213	32814	19791	28036	21422	29055	<0.0001
Inpatient	63503	56674	9502	17911	54001	29982	<0.0001
Emergency	3842	3133	1123	1670	2719	2048	<0.0001
Drugs	27868	18081	10981	20896	16887	20365	<0.0001
Total	137030	108349	42034	66089	94997	76431	<0.0001

CCI, Charlson comorbidity index; *N*, number of subjects; NT$, New Taiwan dollars; SD, standard deviation.

### 3.2. Healthcare costs associated with gMG

The total mean all-cause cost of healthcare in cases who used healthcare resources in 2019 was NT$ 135219.23 (SD 224174.16) (US$ 4455.57, SD 7386.7), versus NT$ 40904.12 (SD 106625.26) (US$ 1347.82 SD 3513.38) in comparators (Table 2). Costs of inpatient cases contributed most to the total raw all-cause cost of healthcare in both cases and comparators, followed by outpatient episodes, drug treatments, and ED visits.

In the regression analysis, the mean difference in healthcare costs between cases and comparators in 2019 was NT$ 94997 (SD 76431) (US$ 3133, SD 2521) (*p* < 0.0001) (Table 3). The difference between healthcare costs was significantly higher in cases compared with comparators for all categories (outpatient, inpatient, ED, and drugs, *p* < 0.0001 for all). The mean cost of inpatient stays in 2019 was NT$ 54001 (SD 29982) (US$ 1781, SD 989) more for patients with gMG than comparators. Outpatient visits cost NT$ 21422 (SD 29055) (US$ 707, SD 958) more, and drug treatments cost NT$ 16887 (SD 20363) (US$ 557, SD 671) more for cases than comparators.

### 3.3. Change in employment status

Significantly more cases than comparators underwent a change in employment status during 2019. Among the 804 cases who were employed in 2019, 106 (13.18%) became either unemployed (dependents) or transferred to a lower income level insurance category, compared with 266 out of 3506 (7.59%) comparators (*p* < 0.0001) (Table 4). Women with gMG were more likely to become unemployed than female comparators, whereas more men with gMG remained employed but transferred to a lower employment category compared with male comparators.

**Table 4:** Cases and comparators (<65 years of age in 2019) who changed employment status during 2019.

Employment category	Cases^a^ (*n* = 804)		Comparators^b^ (*n* = 3506)		*p*
	*n*	%	*n*	%	
Became unemployed (dependent) in 2019	48	5.97	99	2.82	<0.0001
Male	13	3.48	44	2.84	0.5169
Female	35	8.14	55	2.81	<0.0001
Transferred to lower employment categories in 2019	58	7.21	167	4.76	0.0048
Male	28	7.49	66	4.26	0.0095
Female	30	6.98	101	5.16	0.1337
Total	106	13.18	266	7.59	<0.0001
Male	41	10.96	110	7.11	0.0129
Female	65	15.12	156	7.97	<0.0001

a 374 males and 430 females.

b 1548 males and 1,958 females.

## 4. Discussion

We used the NHIRD to identify HRU and costs associated with gMG in 2019. Over the 12 months study period, HRU and associated healthcare costs were significantly higher in cases than a cohort of matched comparators without gMG. While this difference was mainly driven by inpatient costs, outpatient episodes and drug treatments were also substantially higher in cases than comparators, reflecting the chronicity of the disease, the need for life-long treatment, and the costs associated with management of acute episodes such as myasthenic crisis.

We found that over a 12 months period, 13% of employed patients with gMG changed their employment status, of whom 6% became unemployed and classified as dependents in the NHI. This high rate of attrition from the workforce suggests a substantial burden of disease in some patients that negatively impacts their ability to work. Women were impacted more than men, and became unemployed at twice the rate of men.

Few studies have assessed the costs associated with MG in Asia (8). In one hospital-based study in India, the median annual direct cost associated with MG was USD$ 680 but the range of direct costs varied almost 100-fold, from USD$ 67.6 to $6644. There were 21 patients (out of 66) who required intensive care admission for myasthenic crisis. IVIg and plasma exchange were major determinants of cost, and higher costs were statistically associated with disease severity, myasthenic crisis, mechanical ventilation, hospitalization, and intensive care admission (11).

A study in China reported that, in patients hospitalized for MG, the median length of hospital stay was 8 days (IQR 4–15) at a cost of approximately USD$ 1037 (IQR 493–2925). For patients admitted to hospital for myasthenic crisis, the median length of hospital stay was 14–15 days, at a cost of approximately USD$ 3521 (12).

The costs associated with the inpatient management of MG in the United States increased by 13-fold between 2003 and 2013, attributed in part to a new requirement for IVIg to be administered as an inpatient, but mainly to changes in physician decision-making toward admission (13). A cohort study using propensity score matching reported that the annual mean total healthcare costs were 4.5-fold higher in patients with MG than controls. Mean annual pharmacy costs (excluding inpatient costs) were almost 15-fold higher, mainly due to the administration of IVIg during home care. Services administered at home accounted for 23% of the total cost in cases versus 1% in controls. Repeated use of IVIg was a major cost driver (14). In another US-based study, IVIg accounted for 85% of MG-related pharmacy costs, non-steroid immunosuppressives for 9.3%, cholinesterase inhibitors for 5.7%, and steroids for 0.2% (15).

The available evidence suggests that the costs associated with the management of MG vary widely according to physician thresholds for hospitalization and accepted (or insured) medical practices such as administration of IVIg at home, as an outpatient, or as an inpatient (8). The extreme range of HRU and costs in patients with MG reflects that some patients remain stable on oral medication, while others requiring IVIg, plasmapheresis, and hospital admission for the treatment of myasthenia crisis incur much higher HRU and associated costs. The availability of improved treatments that prevent severe gMG could therefore have significant impacts on HRU and costs.

The strengths of our study are the use of the population-based NHIRD that allows comprehensive data capture, including employment status, across the entire population, and the use of recent data that provide a contemporary picture of the healthcare burden associated with gMG in Taiwan. Potential limitations are the absence of a specific ICD code for gMG, which we accounted for by using a combination of hospitalization and treatment criteria. The absence of clinical information in the claims database prevented the identification of patient subgroups which would have allowed a more nuanced understanding of the HRU associated with gMG. Patients with milder forms of gMG not requiring hospitalization in 2010 were not captured in our analysis, suggesting that our results may underestimate the true cost of gMG in Taiwan. We were unable to assess indirect healthcare costs but used change in employment status as an indicator of reduced quality of life.

In conclusion, our study shows that gMG presents a substantial burden on HRU and healthcare costs in Taiwan that exceeds that in individuals without gMG. A high attrition rate from full-time employment suggests additional societal and economic costs that my go unaccounted for in health technology assessments. Improved treatment for MG is needed to alleviate the burden of disease on individuals, healthcare systems, and economies.

## Data availability statement

Publicly available datasets were analyzed in this study. This data can be found here: the data underlying this study are from the NHIRD which has been transferred to the HWDC. The Taiwan government prohibits release of the NHI claims dataset to the public domain. Interested researchers can obtain the data through formal application to the HWDC, Department of Statistics, Ministry of Health and Welfare, Taiwan (http://dep.mohw.gov.tw/DOS/np-2497-113.html).

## Ethics statement

Ethical review and approval was not required for the study on human participants in accordance with the local legislation and institutional requirements. Written informed consent for participation was not required for this study in accordance with the national legislation and the institutional requirements.

## Author contributions

S-PS: software, formal analysis, data curation, visualisation, and writing—review and editing. KH: project administration and writing—review and editing. YL: conceptualisation, methodology, supervision, and writing—review and editing. C-CY: writing—review and editing. C-HT: study conceptualisation, methodology, data curation, formal analysis, and writing—review and editing. All authors contributed to the article and approved the submitted version.

## Funding

This work was supported by Janssen Asia Pacific, a division of Johnson & Johnson Pte Ltd.

## Conflict of interest

KH is an employee of Janssen Asia Pacific and YL is an employee of Epidemiology, Office of the Chief Medical Officer, Johnson & Johnson. KH and YL hold stock in Johnson & Johnson.

The remaining authors declare that the research was conducted in the absence of any commercial or financial relationships that could be construed as a potential conflict of interest.

## Publisher’s note

All claims expressed in this article are solely those of the authors and do not necessarily represent those of their affiliated organizations, or those of the publisher, the editors and the reviewers. Any product that may be evaluated in this article, or claim that may be made by its manufacturer, is not guaranteed or endorsed by the publisher.
